# Adaptation and development of software simulation methodologies for cardiovascular engineering: present and future challenges from an end-user perspective

**DOI:** 10.1098/rsta.2009.0052

**Published:** 2009-07-13

**Authors:** V. Díaz-Zuccarini, A.J. Narracott, G. Burriesci, C. Zervides, D. Rafiroiu, D. Jones, D.R. Hose, P.V. Lawford

**Affiliations:** 1 Cardiovascular Engineering and Medical Devices Group, Department of Mechanical Engineering, University College LondonTorrington Place, London WC1 7JE, UK; 2 Academic Unit of Medical Physics, I Floor, Royal Hallamshire Hospital, University of SheffieldGlossop Road, Sheffield S10 2JF, UK; 3 Electrical Engineering Department/Bioengineering Centre, Technical University of Cluj-Napoca400020 Cluj-Napoca, Romania; 4 Department of Medical Physics, Rotherham District General HospitalRotherham S60 2UD, UK

**Keywords:** cardiovascular modelling and simulation, simulation software, virtual physiological human, patient-specific simulations, multi-scale modelling, biological modelling

## Abstract

This paper describes the use of diverse software tools in cardiovascular applications. These tools were primarily developed in the field of engineering and the applications presented push the boundaries of the software to address events related to venous and arterial valve closure, exploration of dynamic boundary conditions or the inclusion of multi-scale boundary conditions from protein to organ levels. The future of cardiovascular research and the challenges that modellers and clinicians face from validation to clinical uptake are discussed from an end-user perspective.

## 1. The predictive paradigm for the treatment of cardiovascular disease

Clinicians use a number of different tests to determine the nature of a medical condition and then plan a treatment/intervention based upon experience. Success may be defined in a number of ways depending on the nature of the treatment; it may be the ability to regain certain bodily functions, or to increase life expectancy. The range of inter-patient variability, particularly when disease is present, represents a significant challenge to determining appropriate treatment and a reliance on statistical methods based on average behaviour can result in poor predictive power for individual patients.

It is interesting to compare medical practice with engineering. Both disciplines are based on problem solving. In engineering, there is an attempt to predict accurately the performance of a product or procedure. The entire design process is based upon predicted outcomes. Very often a number of criteria must be satisfied simultaneously and sophisticated computer and analysis technologies are employed to accomplish this. By contrast, owing to the complexity and inherent variability of the underlying biological behaviour, medicine has tended to adopt a semi-empirical approach, with observations from clinical studies interpreted in combination with detailed *in vitro* analysis of individual components of the system. An exciting option that is currently being investigated is the possibility of combining these two different approaches. Recent advances in information and communication technology have enabled predictive engineering methodologies to be integrated within the medical environment. A future can be envisaged where technology is adapted to individual patients, with appropriate treatment conceived to suit a set of specific and unique needs. This integrative vision of human physiology, in combination with the advances in computational modelling and simulation, will help unravel the systemic nature governing many of the physical manifestations of disease.

Cardiovascular engineering is one of the success stories in bioengineering. The application of mathematical modelling in cardiovascular physiology gained prominence in the 1950s with Hodgkin and Huxley's successful prediction of the speed of action potential propagation along a nerve fibre from cable theory, coupled to models of ion channel conduction and gating kinetics (Hodgkin & Huxley 1952; Noble 1962). Other early successes in applying techniques taken from the physical sciences to cardiovascular physiology were engineering analyses of blood flow in arteries using computational fluid dynamics (CFD). These were later coupled with finite-element (FE) analyses to model the structural behaviour of tissue. Until fairly recently, simulation of arterial blood flow involved the use of very simple, idealized models and the relevance to medical practice was very limited. More realistic representations of the arterial system (Jones *et al.* 2004*a*,*b*) that can be used to simulate pre-operative, diseased configurations and to analyse post-operative outcomes are now employed (Migliavacca *et al.* 2005). ‘Predictive medicine’ in which patient-specific computer models are used to evaluate the efficacy of a number of possible treatments and to plan and design the optimal intervention based on outcome prediction is now a possibility. It is clear that, with current technology and sufficient effort, the immediate consequences of an intervention might be predicted. The effectiveness of a bypass graft configuration can be assessed with reference to the redistribution of blood flow, for example. Of even greater importance is the prediction of the implications of intervention after time has elapsed: one month; three months; six months; one year; two years; or more.

Two specific examples of cardiovascular engineering applications are the study of coronary stent implantation and the design analysis of prosthetic heart valves. Stents are devices that re-establish and maintain the patency of an artery after development of atherosclerotic plaque and narrowing of the vessel lumen. Approximately 1 out of 10 such procedures ‘fail’, not structurally, but due to abnormal cell proliferation within the vessel wall leading to intimal thickening, restenosis and thrombus formation. In non-technical terms, the artery recloses, restricting blood flow. These phenomena involve chemical and cellular responses to mechanical stimuli, such as strain in the vessel wall and wall shear stress induced by the intervention and redistribution of blood flow. The exciting prospect is that these can be represented mathematically; researchers are beginning to develop models of these phenomena. Growth and adaptation models are envisioned not only for the response of biological tissues to external intervention but, additionally, for the evolution of the disease process, in this case, restenosis (Evans *et al.* 2008).

Research related to valve replacement and repair is of significant clinical interest. While existing mechanical heart valves (MHVs) and biological valves (BVs) are used routinely, there are still important issues to solve regarding the inherent thrombogenecity of MHVs and the durability of BVs. In addition, new endovascular techniques for percutaneous heart valve implantation are being developed to avoid open heart surgery (Bonhoeffer *et al.* 2000) and the design and implementation of these devices is in an on-going developmental phase. There is a clear role for modelling and simulation in the study of both existing and novel devices, and significant work related to the assessment and behaviour of these valves under patient-specific conditions and with reference to pharmacological intervention remains to be carried out.

Ultimately, a holistic representation encompassing the complex interactions between the heart, vasculature and the systemic response to the changing physiological environment is needed. This requires patient-specific models of the heart to consider all the integrated elements of the system. Impressive and detailed models of the heart have been developed already (Hunter *et al.* 2001) but, due to their significant computational requirements, they are not as yet applicable in the context of the rapid or real-time simulation that is often demanded for diagnostic purposes. There remains a place for simpler representations of the fundamental mechanisms describing the heart beyond the ‘elastance’ model, well known to cardiovascular scientists, which became a paradigm in cardiovascular engineering. With regards to the elastance concept, while engineers and physicists tried to explain cardiovascular function in mathematical terms that had little meaning to the ordinary physiologist, a real scientific revolution took place in a totally different arena. Ideas and techniques developed for studying the molecular foundations of physiology captured the imagination of cardiovascular scientists. Within this new framework, multi-scale and multi-physics modelling approaches seem to be the way forwards but the future remains open in this area.

In this paper, we present the application of a number of diverse software tools in cardiovascular modelling and simulation from the end-user's perspective. While these tools essentially belong to the field of engineering, the applications presented push the boundaries of the available software to address specific events related to venous and arterial valve closure, the exploration of dynamic boundary conditions or the inclusion of multi-scale boundary conditions from the level of the protein to the organ.

## 2. Cardiovascular modelling and simulation today: software development and methodologies

The key to successful computational physiology is the capture of structure–function relationships in a computationally efficient manner. This approach requires models that are fundamentally different from those in the world of engineering (Crampin *et al.* 2003). Development of multi-scale models that take into account the underlying biological mechanisms and interface local three-dimensional models with systemic models has the potential to improve substantially the current simulation framework based on established software packages. Owing to their complexity, the behaviour of such models is normally studied in isolation. This is far from representative of the physiological situation. One solution is to provide boundary conditions and an appropriate environment with the same detail as the model itself. This issue raises a number of questions: is this level of complexity and its associated computational overhead necessary; do we need highly detailed models of the environment in order to reproduce, or understand, the behaviour of a system; and is this currently possible in terms of computational effort?

### (a) Quantitative versus qualitative results

The question of a qualitative versus a quantitative approach is at the core of the diagnosis paradigm. Although it is generally understood that medicine is far from an exact science, it is still necessary to obtain quantitative data within a certain level of accuracy in order to provide reliable tools for diagnosis. Diagnosis is based on knowledge extracted from a multitude of sources, including quantitative tools, although there are many subjective factors in play. Knowledge gained can be influenced by context and the interaction between the doctor and the patient. Qualitative results are often useful to help interpret quantitative findings as they are extremely important for understanding ‘why’ questions.

Quantification is often difficult as there is significant variability from patient to patient, prompting a population-based approach. When applied to medicine, physical findings which attempt to answer ‘why’ questions must have individual relevance, as diagnosis must be made on an individual basis taking into account specific patient characteristics. It becomes evident that for precise results, we will need detailed quantitative models. This point is crucial. Not only do we need sophisticated tools that are reliable from a numerical point of view, but we also need adaptable tools able to cope with multi-physics and multi-scale problems ranging from molecular to physiological levels. This increasing level of complexity generates a cost, in terms of computational resources, that is unavailable to many researchers. In-house tools must be developed, maintained and updated, or the cardiovascular scientist must rely on commercial software, adapting it to specific needs. In §2*b*, we present models employing customized software, for diverse applications. In many cases, this required close interaction between researchers and software developers. The role of boundary conditions is also discussed.

### (b) Specific applications and collaboration with software developers

#### (i) Development of fluid–structure interaction tools, the Bloodsim project and applications

Many cardiovascular problems involve significant interaction between fluid (primarily blood) and solid (blood vessel walls, heart valve leaflets) components. With advances in computational hardware, numerical solution of fluid–structure interaction (FSI) has recently become achievable and applied to cardiovascular problems (Krafczyk *et al.* 2001; De Hart *et al.* 2003; Griffith *et al.* 2007). Between 1998 and 2001, the Bloodsim project developed tools to provide an external coupling between the commercial solvers ANSYS (structural, FE) and CFX (fluid, finite volume) to solve such problems. Successful implementation of the coupling scheme was achieved through the inclusion of CFX as a partner within the project consortium, allowing access to the code at a low level and providing significant solver development experience. (It is of interest to note that, following acquisition of CFX by ANSYS in 2003, a similar approach to coupling between the two codes was implemented as an ‘out-of-the-box’ feature of the ANSYS multi-field analysis option.) An attraction of coupling existing mature solvers is that they contain optimized specialized features, including nonlinear structural materials, large deformations, contact and advanced fluid models. Applications of the Bloodsim FSI code included an examination of flow local to a stent geometry deployed within a uniform elastic tube (Hose *et al.* 2003). This analysis included contact of the stent with the tube wall during deployment, resulting in a complex internal fluid domain. The Bloodsim approach was also applied to the analysis of forces acting on a prosthetic heart valve at closure (Hose *et al.* 2006). In this example, the rigid body solution of the coupling scheme was employed. When considering mechanical valves, it is advantageous to neglect the structural response of the valve leaflet and consider it as a rigid body. This allows the fundamental dynamics of leaflet motion and the forces due to sudden arrest of the local fluid at closure to be determined without the solution of the full structural response. Coupling is still required between the calculated rigid body motion and the update of the fluid mesh geometry and local fluid forces.

#### (ii) Simulation of natural valves and investigation of deep vein thrombosis

The use of independent solvers that iteratively pass interface values of either displacement or pressure fields to achieve a coupled solution has been shown to be effective for a range of problems. However, for highly flexible structures, it is often difficult to obtain a stable response using such schemes, a particular example being native cardiac valves. With the exception of a recent lattice–Boltzmann simulation (Buxton & Clarke 2006), little attention has been paid to the valves in the venous system. Given the potential involvement of venous valve haemodynamics in the development of deep vein thrombosis (Malone & Agutter 2006), this is an area that warrants more detailed investigation.

**Figure 1:**
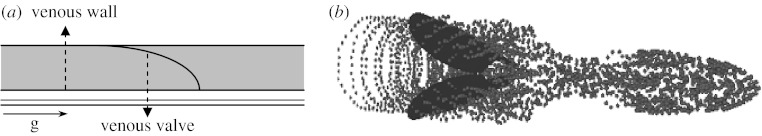
(*a*) The fluid was represented using eight-node solid nodes and the vein wall and venous valve were represented by four-node shell elements (LS-DYNA model of vein and valve). The direction of gravity is indicated. (*b*) Flow was established using a parabolic inlet condition representing the input flow of the femoral vein and a zero output pressure condition to allow unimpeded flow through the valve (seeded particles illustrating evolution of flow local to valve structure).

A three-dimensional venous valve model using the commercial code LS-DYNA (v. 971) was developed. LS-DYNA is an explicit FE program used in the analysis of nonlinear dynamic responses of three-dimensional structures. A particular attraction, and the reason for its selection for simulating venous valves, is the relative stability of its approach to fluid–solid interaction when applied to very flexible structures (Carmody *et al.* 2006; Kunzelman *et al.* 2007). The model geometry is shown in figure 1. This implementation requires additional fluid elements to be defined external to the vessel, allowing fluid to move into these elements as the solution progresses.

For this application, the model geometry was generated using ANSYS classic. Ansys Parametric Design Language was then used to output the model geometry in a format that could be directly imported into the Primer pre-processor (Ove/Arup). Once boundary conditions were applied, the model was solved within LS-DYNA and the results interpreted using the d3plot post-processor (Ove/Arup).

Ensight v. 8.2 (CEI) software was used for extended visualization facilities, movie creation, image capturing and particle tracking. The latter was particularly appropriate for this application, allowing the development of methods to quantify the ‘washout’ of blood particles in the regions of interest, as shown in figure 1. A primary aim was to determine the effects of gravitational loading on valve haemodynamics; the results showed that the application of gravity helps to move blood from regions of stasis. Validation of the method was obtained by comparing the results of the three-dimensional model with theoretical solutions and with the results obtained from published one-dimensional models (Zervides 2008; Zervides *et al.* 2008*a*,*b*). The potential of these models is enormous. Further applications include patient-specific simulations to examine the effect of compression therapies and pharmacological prophylaxis and the development of prosthetic venous valve implants.

#### (iii) A detailed representation of the arterial system for patient-specific simulations

**Figure 2:**
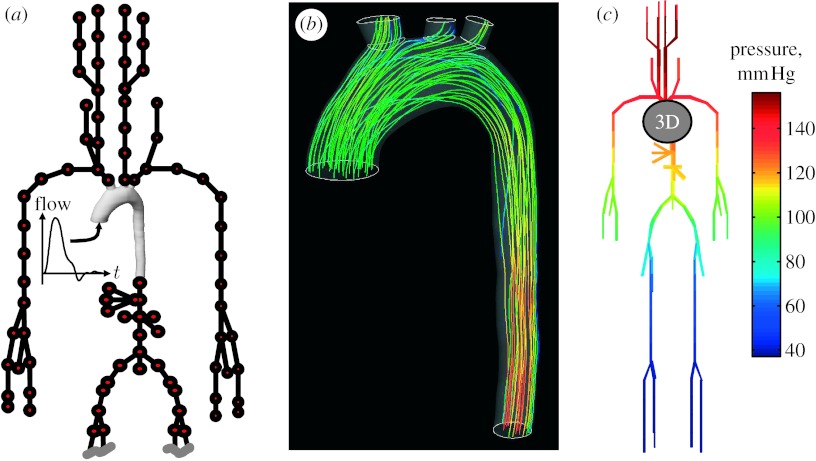
(*a*) The inlet flow to the aorta model was specified using data from a flow-sensitive fast-field magnetic resonance echo sequence acquired in the same session as the anatomical scan. The outlets of the model were coupled to linear compartmental models that represent the downstream circulatory system (three-dimensional mesh coupled to downstream compartments). (*b*) CFD simulation of the three-dimensional aorta model is performed using CFX. Reference mesh coupled to the compartment models and results for the three-dimensional model and compartment systems (three-dimensional flow solution at peak systolic flow). (*c*) The solution of the compartment models allows the pressure and flow at downstream locations in the circulatory system to be determined (compartment solution at peak systolic flow). Also, the compartment models provide improved boundary conditions for the three-dimensional model.

If it were possible to simulate the entire human body, one could envisage a completely closed fluid model, including the heart, arteries, capillaries and veins. However, to model this as a single three-dimensional model is impractical. The patient scan data required to obtain the geometry is often confined to a particular region of the body. Thus, at some point, the fluid model must be truncated to give inlets and outlets where boundary conditions must be specified. In the simplest case, flow rates or pressures are imposed directly at the boundaries. Alternatively, a simplified representation of the remainder of the circulatory system can be coupled to the boundaries. This reduced model would typically be based upon standardized data from the general population rather than being for a specific patient. At its least complex, the model could be a simple resistance or a Windkessel-type model. The problem of coupling together models of differing complexity has been considered by Formaggia *et al.* (2001) coupling a three-dimensional fluid model with a solid model wall to a nonlinear one-dimensional model at the outlet. This was found to be an effective way of reducing spurious wave reflections at the outlet boundary of the three-dimensional model. The coupling of a three-dimensional model to a zero-dimensional compartment model, using state variables to describe the compartment model, has been analysed by Quarteroni & Veneziani (2003). This approach in the clinical application of paediatric cardiac surgery is used by Migliavacca *et al.* (2005). A scheme for coupling a three-dimensional model to a digital implementation of the Westerhof model (in both frequency and time domains; Westerhof *et al.* 1969) has been implemented. The frequency domain analysis simply involves adding up complex impedances in series or parallel until the ‘root impedance’ of the whole branching tree is determined at the desired frequency. One method of obtaining the time domain response would be to perform an inverse Fourier transform of the frequency response. However, this approach is applicable only to repetitive signals, and does not easily lend itself to determination of the pressures and flows throughout the model. Instead, a model of the Westerhof circuit using a state-space approach was created. The results of a simulation of this type are shown in figure 2.

For patient-specific simulation, it is important that simulations can be performed on a routine basis in the clinical setting. In this context, the user interaction required to create the CFD model must be minimized, automating the mesh generation process as far as possible. Furthermore, the high-performance computing (HPC) required for transient CFD simulation must be accessible, possibly using grid technology (Jones *et al.* 2004*a*,*b*).

#### (iv) Three-dimensional simulation with biologically oriented boundary conditions: the C-CARES project

Section 2*b*(iii) illustrates the realistic representations of the arterial tree for patient-specific simulations (Jones *et al.* 2004*a*,*b*) describing appropriate boundary conditions for the afterload, using patient-specific data (length of vessels, diameter, etc.). However, the cardiovascular system is a coupled heart–arterial network system, and thus both the pumping and load systems must be described correctly. Dynamic boundary conditions, described in terms of pressure and flow, are certainly better than imposed input and output conditions from a haemodynamic point of view. This section illustrates how this idea was developed further. A coupled system of a St Jude MHV and biologically based boundary conditions of the pumping system is presented (Rafiroiu *et al.* 2008), taking into account fundamental biochemical details of cardiac contraction. This kind of approach, which has already been presented in the context of an idealized valve (Díaz-Zuccarini *et al.* 2007), can provide insight into the underlying biological mechanisms of contraction at different levels, from the protein to the organ. A multi-physics and multi-scale lumped-parameter model of the left ventricle was used as a boundary condition for a three-dimensional model of a St Jude valve in mitral position and was implemented in CFX by developing specific Fortran subroutines, making use of advanced user tools such as junction boxes and subroutines within the CFX software (Díaz-Zuccarini *et al.* 2008). Valve geometry was obtained by laser scanning thus ensuring that the fine detail of the design around the hinges was captured. The geometry was imported into ANSYS Workbench and the FSI simulation performed using CFX v. 10.0. Collaboration with the software developers was required to develop semi-automatic remeshing tools, taking advantage of meshing interpolation techniques and the ANSYS Workbench–CFX interface. The extent to which these simulations can be parallelized remains to be investigated and it certainly represents a challenge. One possible application of this methodology is the exploration of the effect of pharmacological intervention on the behaviour of medical devices and implants. This is a key issue since patients undergoing this type of surgery normally receive medication for life; information could be provided on possible outcomes and alternative treatments.

#### (v) Design of percutaneous heart valves and the relevance of anatomical data for patient-specific purposes

Valve replacement by percutaneous transcatheter implantation is a novel procedure that allows a prosthetic valve to be implanted by delivering the valve in a stent into the aorta via a catheter inserted through an artery in the groin. This technique eliminates some of the risk associated with conventional surgery, providing an alternative treatment for patients currently excluded from conventional open-heart operations. First clinical experiences using this approach indicate that the technique is both feasible and promising (Ghanbari *et al.* 2008).

Numerical simulation of percutaneous valves is challenging. The valve has to be collapsed to a small diameter for catheter delivery and then expanded to the operative configuration within the aorta. Modelling this process requires large deformations of highly nonlinear materials, for both balloon-expandable stents and self-expanding hyperelastic stents. The valve component is typically manufactured from a biological soft tissue or hyperelastic polymer, and all the difficulties mentioned in the context of simulation of natural valves apply. The implicit commercial package MSC.Marc was chosen to perform the simulation of both crimping and releasing of the valve. This code is efficient when dealing with nonlinearities, including severe contact problems. Moreover, it contains a set of material models suitable for describing the mechanical and thermo-mechanical behaviour of superelastic and memory-shape alloys such as nitinol, commonly used in self-expanding valves (Auricchio *et al.* 1997). The effect of blood flow during valve release required FSI analyses with LS-DYNA.

We envisage that numerical simulation, especially patient-specific and predictive modelling, will have an increasingly important role in the development of percutaneous valves, since functional performance is strongly dependent on the specific anatomy of the patient and on their material properties. It is essential to be able to ensure that the minimal valve requirements are guaranteed for a specific patient, and can be maintained for a sufficient time as native structures change due to age-related physiological modification or as a response to the treatment.

## 3. Current limitations and future challenges

There are three primary issues. First, this approach is computationally intensive, pushing the boundaries of what can be achieved with commercial software. From a numerical point of view, the multi-scale nature of the applications, and time-varying and nonlinear characteristics of the boundary conditions, in combination with the coupling methodologies and discretization techniques used to define them, often requires small time steps to achieve convergence. This issue could prove critical as the simulation of long physical time scales is constrained by the extent of the physical domain, mesh refinement requirements and fluid behaviour, among other things. HPC simulation, possibly using grid technology, must be accessible.

Second, a conceptual problem arises in understanding the biology behind the problem and extracting the most relevant information to define and implement the computational models. This is particularly relevant to patient-specific modelling in the context of routine simulation in a clinical environment. A comprehensive set of parameters for the ‘patient-specific’ model must be defined at different physical and biological scales and account must be taken of individual inter-variability and ageing processes. A question then must be asked: what is patient-specific modelling and in which context? This is intimately linked to clinical uptake as discussed briefly below. Several related problems now emerge. The biological mechanisms of different processes need to be understood separately, each described in different terms and relevant parameters and mechanisms identified. These models then have to be translated mathematically using independent levels of detail while ensuring that the translation process remains biologically and physically relevant and coherent. This flexibility must be taken into account by software developers and a joint effort is required to develop computational frameworks for multi-science and multi-scale simulation. Recent initiatives (Evans *et al.* 2008) propose a structured way to deal with these problems. These approaches must start to be incorporated in commercial software applications to reach the simulation community at large. Such tools must allow integration of data ranging from the molecular level (spatial scale: 10^−9^ m; time scale: 10^−6^ s) to the organism (spatial scale: 10^0^ m; time scale: 10^9^ s) level (Fenner *et al*. 2008) and, in combination with the existing grid infrastructure and other HPC platforms, to apply this understanding to the clinic. A multi-scale modelling framework could provide a more rational basis for diagnosis and treatment than currently exists. It is not obvious how to achieve continuity in models and simulations across biological levels, each of which is normally governed by specific rules and assumptions (DG INFSO & DG JRC 2005).

Last but not least, there is a long journey between simulation and treatment. Clinical factors such as the severity of the disease, availability of treatment, and effectiveness and accessibility of screening and surveillance methods will influence clinical uptake. Technical accuracy (analytic validity); predictive power (clinical validity); potential for improving healthcare and health outcomes (clinical usage); and ethical, legal and social implications (Shah 2004) will all be important. Clinical applications will have to meet rigid constraints. As recent initiatives, such as the VPH, have placed a strong emphasis on industrial and clinical applications, these are some of the challenges that such initiatives will certainly confront.

## 4. Conclusions: the future of cardiovascular research

There are lessons embedded in the history of cardiovascular physiology. We need to update the definition of physiology to include computational physiology as an integral part of the process. There is a strong need for specialist understanding of the behaviour of the parts as these are required to illuminate the behaviour of the whole. But this process is bidirectional. A complete understanding of cardiovascular function requires input from biophysics, biochemistry, pharmacology, structural biology, genetics, bioengineering, mathematics and other fields. Improving fundamental understanding is a common goal that is rarely achieved at present. We need to put this integrated knowledge within a context, which is provided by systems biology. The current discipline-based organization of the biomedical, engineering and physical sciences is inconsistent with a revolution in biology that has provided a common conceptual foundation now shared by all disciplines, as evidenced by the current proliferation of multidisciplinary faculties and institutes. We must also train university students to develop a broad view of cardiovascular science that spans from molecular biology to computational physiology with an integration of human biology as a whole. An effort will be needed at the academic level to break down the barriers created by the traditional disciplinary approaches. We also need to take research out of academic circles and to translate cardiovascular engineering into clinical applications, with patient-specific treatment as the ultimate long-term goal. Finally, we need greater integration with software developers to guide the development of specific tools to model biological systems. This fast-growing area requires the cardiovascular engineer to take on a role that focuses on the modelling, simulation and analysis of cardiovascular disease. Computational physiology has been identified by Sun and competitors as the ‘next big thing’ (Malcolm 2003); thus we could argue quite confidently that an increasing market for these complex software applications is about to explode. This synergy will certainly facilitate the translation of basic research into clinical strategies for care of patients with cardiovascular disease. With necessity as a driving force, a multi-scale approach from the molecular and cellular level up to the organ and system levels is emerging. The solution to this problem is far from simple and will certainly require input from mathematicians, physicists, computer scientists, engineers and biologists.
